# Outcomes of Meniscal Repair in Patients Aged 40 and Above: A Systematic Review

**DOI:** 10.3390/jcm12216922

**Published:** 2023-11-03

**Authors:** Rawan Jaibaji, Faisal Khaleel, Monketh Jaibaji, Andrea Volpin

**Affiliations:** 1 Imperial College NHS Healthcare Trust, London W2 1NY, UK; rawan.jaibaji1@nhs.net; 2 Michael G. DeGroote School of Medicine, McMaster University, Hamilton, ON L8S 4L8, Canada; khaleef@mcmaster.ca; 3 Health Education North East England, Newcastle upon Tyne NE15 8NY, UK; monketh.jaibaji@nhs.net; 4 NHS Grampian, Aberdeen AB15 6RE, UK

**Keywords:** meniscus repair, knee surgery, knee arthroscopy

## Abstract

Purpose: Meniscal injuries are increasingly common in older age groups. Age is often cited as a contraindication to undergoing meniscal repair due to concerns regarding failure rates. There has recently, however, been an increasing shift towards repair in older populations. The purpose of this study was to review outcomes of meniscal repair in patients over the age of 40. Methods: A systematic search of the following databases was conducted of PubMed, SCOPUS, Web of Science, and Cochrane Library to identify studies reporting failure rates of patients over 40 with meniscal injuries undergoing repair. The definition of meniscus failure was noted for each study evaluated in this systematic review. Further data surrounding clinical and radiological outcomes were recorded and evaluated, when available. Results: Thirteen studies were included in this review, encompassing a total of 316 meniscal repairs in patients over the age of 40 years. The overall failure rate was found to be 15.5% (49/316) (range 0–33.3%). There was no difference in the failure rate in those over 40 vs. under 40, and the two groups had equivalent functional outcomes. Conclusions: Age should not be considered a contra-indication for meniscal repair. Appropriately selected older patients can have acceptably low failure rates with meniscal repair and similar functional outcomes to those under the age of forty. Meniscal repairs in those over 40 achieved better functional outcomes than patients of the same age group who underwent meniscectomy.

## 1. Introduction and Background

Meniscal tears are a common injury, with the estimated annual incidence of 61 per 100,000 individuals [1]. The menisci function to increase stability of the femorotibial articulation, distribute axial load, and have a role in the proprioception and lubrication of the knee joint. Damage or removal of the meniscus leads to degenerative changes within the tibiofemoral joint. Meniscectomy (total and partial) was the main-stay surgical treatment for meniscal tears. However, the increased recognition of the biomechanical importance of the menisci has shifted the paradigm towards preservation surgery, especially in younger and more active patients. In older patients, where the tears are more often degenerative in nature, partial meniscectomy remains a common intervention, whereas repair is more likely to be attempted in the younger population [2,3].

Meniscal tears of an extent that requires treatment are 2–3 times more common in patients over 40 [4]. There has been a trend towards meniscal repair over partial meniscectomy [5]. This is mainly attributed to the positive patient outcomes in the case of meniscal repair for younger and higher-demand patients. There are, however, less studies examining the functional outcomes associated with meniscal repair in older patients [6,7]. There is an increased recognition that the repair failure rate in this age group may not be high enough to justify opting for a partial meniscectomy. Moreover, accelerating degenerative changes in this age group may have significant health and socioeconomic consequences further down the line.

A systematic review conducted in 2018 [3] aimed to report the failure rate of meniscus repair in patients over 40; it concluded that there is insufficient evidence that age is an independent contraindication to meniscus repair and that the reported failure rate was comparable to that in the general population. Since this study was published, more data have become available, including more studies reporting functional outcomes, which were not included in this review. The purpose of this review is to provide an updated evaluation of the failure rates in meniscus repairs in patients over the age of 40 years. As a secondary outcome, we aimed to evaluate functional outcomes in this cohort.

## 2. Methods

**Figure 1:**
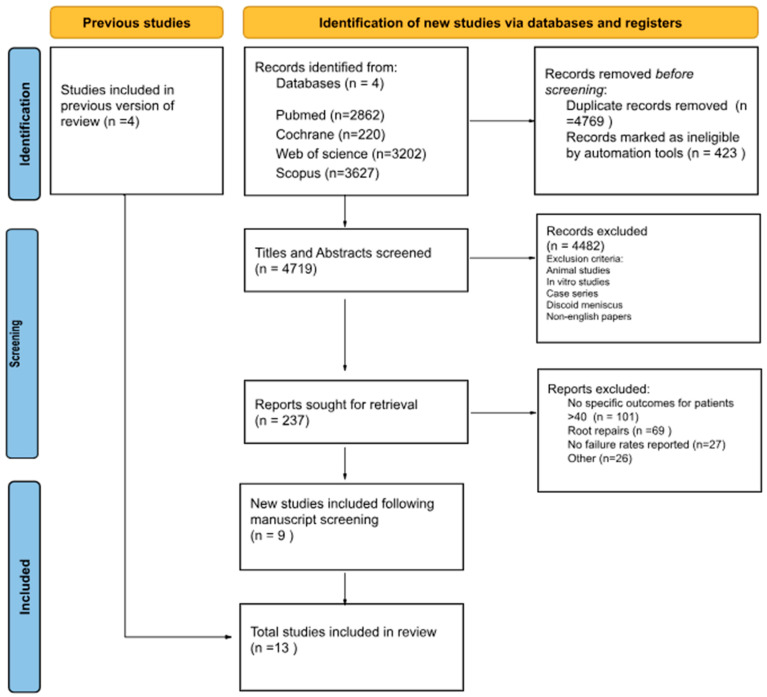
PRISMA flow diagram for systematic review of failure rates of meniscus repairs in patients over the age of 40 years.

A systematic review in accordance with PRISMA guidelines [8] was conducted on the following databases: PubMed, SCOPUS, Web of Science, and Cochrane Library. The following search terms were used (meniscus or meniscal AND repair and (outcome* or result or measure*). A total of 9911 studies resulted from the initial search. Duplicates were removed, and 4719 titles and abstracts were independently screened by the first two authors. Following title and abstract screening by the first two authors, the full manuscripts were retrieved and independently screened by the first two authors in accordance with the inclusion criteria. Disagreements were discussed and resolved with the consensus of all authors. The results of the search are shown in Figure 1. The following exclusion criteria were applied: (1) studies with patients exclusively under the age of 40, or did not present the data for the 40 years old and above demographic independently; (2) in vitro studies; (3) meniscal transplant studies; (4) discoid menisci; (5) meniscal root repairs; (6) case reports; (7) database analysis; (8) studies published prior to the year 2000. With regard to age, 40 years was selected as the cut off through consensus amongst the authors as it reflects the cut-off most used in the published literature.

The primary outcome was the failure rate of meniscal repair. Failure rates were recorded according to what the individual studies defined as failure. Further data surrounding clinical and radiological outcomes were recorded and evaluated, when available. The authors of studies where patients with no specific results for those over 40 were contacted to see if these data could be provided. If they could not, the manuscript was not included in the formal analysis but, where outcomes related to age were cited, they were included in this review where relevant. In studies with overlapping cohorts, the series with the larger number of patients was included. If the numbers were the same, the more recent study was included. Risk of bias was independently assessed using the MINORS criteria [9] by the first two authors.

The protocol of the systematic review was registered with PROSPERO (University of York, York, UK) with the following number: CRD42023434500.

## 3. Statistical Analysis

All statistical tests were performed with weighted data. Analysis of categorical data was performed using cross-tabulation and Fisher’s exact test. Between-group comparison of continuous data was performed using an independent samples *t*-test. Pearson’s rank correlation was used to measure correlation between variables. Data were analyzed using GraphPad Prism version 10.0 (GraphPad Software, Boston, MA, USA). A *p* value of <0.05 was considered significant.

## 4. Results

Thirteen studies were included in this review, encompassing a total of 316 meniscal repairs in patients over the age of 40 years [10,11,12,13,14,15,16,17,18,19,20,21,22]. There were no randomized controlled studies included or studies where data were collected prospectively. One retrospective case series and twelve cohort studies were included. Two studies declared external sources of funding, with either direct or indirect industry support [12,22].

The mean age across all studies was 47.5 years; the median was 48.2 years. The mean follow-up length was 66 months (range 16.5–192). We found no significant correlation between length of follow-up and failure rate (r = 0.198, 95% CI −0.396–0.676, *p* = 0.52).

### 4.1. Failure Rates

The overall failure rate was 15.5% (49/316) (range 0–33.3%) across the 13 studies [10]. Three studies [16,17,23] comparing failure rates between patients above and below the age of 40 years demonstrated a slight increase in failure rate in those over 40 compared to those under 40: 10% (5/50) vs. 6% 8(8/137) (OR 1.79, 95% CI 0.63–5.56, *p* = 0.34). The failure rate in knees that were known to be ACL-deficient was 13% (6/46 knees).

### 4.2. Repair Technique

A total of 187 medial meniscus repairs and 66 lateral repairs were reported, and 5 patients had both menisci repaired. Where the operative techniques were reported, 63 meniscal repairs used an all-inside technique, 130 used inside-out, and 4 used an outside-in technique. In all-inside meniscus repairs, six studies reported the use of a Fast-Fix© system (Smith & Nephew, Andover, MA, USA) [10,11,12,14,22,23], one study reported the used of Biofix Arrow Fixation© [17], one study reported the use of a VIPER© repair system (Arthrex, Naples, FL, USA) [17], and one study reported the use of a Zone Specific II Meniscal Repair System (Linvatec, Largo, FL, USA) [12]. In studies where meniscus repair was performed using the inside-out technique, a Juggerstitch© Meniscal Repair Device and fast-fix system (Smith & Nephew, Andover, MA, USA) were utilized [10,20]. Nakayama et al. also used augmented repair with fibrin clot implantation [20]. Meniscus rasping to promote healing was reported in two studies [10,22]. Table 1 shows the meniscus tear characteristics of patients included in this review when reported. Table 2 is a summary of the studies included.

### 4.3. Patient-Reported Outcome Measures

Eight studies included patient-reported outcome measures in their series [10,11,12,13,14,20,21,22,23]. All eight of these studies included reported postoperative Lysholm scores. Four studies used the IKDC, three studies reported the KOOS score, two studies reported the Tegner activity scale, and one study reported pre- and postoperative VAS scores. The pooled weighted mean postoperative Lysholm score was 85.5. The Lysholm score showed that the meniscus repair in patients over 40 vs. under 40 was 87.6 vs. 85.7, respectively; the data was not reported in a way that allowed for weighted analysis.

Three studies [10,12,13] compared the outcomes of patients over 40 years of age undergoing meniscus repairs versus meniscectomy. Weighted analysis using the independent samples *t*-test of the postoperative Lysholm score showed statistically significant better functional outcomes in the meniscal repair group (86.6 (SD ± 7.75) vs. 66.2 (SD ± 6.87) *p* < 0.0001).

### 4.4. Radiological Outcomes

Three studies [16,20,21] reported the radiological (MRI) outcomes of meniscus repairs according to the Henning Criteria; the weighted mean failure rate was 24% (range 14–33%). One study [12] used postoperative radiographs, comparing outcomes with meniscus repairs and meniscectomy changes, and reported no statistically significant difference.

### 4.5. Postoperative Rehabilitation

Eleven studies reported their postoperative rehabilitation protocol following meniscal repair [10,11,12,14,16,17,18,19,20,22,23]. Non-weight-bearing ranging from two to six weeks was seen in eight studies [10,11,16,18,20,21,22,23], and immediate protective weight-bearing was seen in three studies [12,17,19]. The rehabilitation program consisted of fixed knee extension for 2–3 weeks in three studies [11,18,20] and limitation of flexion to 90 degrees in the remaining studies [10,14,16,17,19,21,22,23].

### 4.6. Methodological Quality

When assessing the studies included in this review using the MINORS criteria [9], all the studies had a clearly stated aim, with adequate follow-up and study endpoints identified. The median value was 14 (IQR 7). Lower scores were most commonly due to lack of a control group and lack of blind evaluation of endpoints.

**Table 2:** Summary of 13 studies included in this systematic review [10,11,12,13,14,15,16,17,18,19,20,21,22].

Author	Year	Level of Evidence	MINORS Criteria Score	Mean Age (years)	Males Included (%)	Mean Follow Up (months)	Number of Repairs	Failure Rate (%)	Criteria for Failure
Ventura et al. [10]	2023	3	20	50.95	14 (63.6)	24	22	4.55	Repeat surgical intervention
Zhu et al. [11]	2022	4	14	57.7	18 (66.7)	52	27	11.11	Barret criteria
Husen et al. [12]	2022	2	20	64.5	12 (60)	39.4	13	23.08	Repeat surgical intervention
Engler et al. [13]	2021	3	21	48.5	11 (52.4)	59	28	17.86	Repeat surgical intervention or surgeon report of failure
Nakayama et al. [20]	2020	4	13	47	20 (83.3)	39.3	24	25	One of the following: (1) development of pain at the joint line associated with motion and/or catching, locking, or swelling; (2) intrameniscal fluid signal along the repair area on follow-up MRI; (3) repair failure confirmed by second-look arthroscopy
Poland et al. [22]	2019	3	21	47.2	28 (50)	60	56	17.86	Repeat surgical intervention
Buyukkuscu et al. [21]	2019	3	13	46.1	23 (69.7)	31.3	33	33.3	Clinical failure: patient symptoms and Barrett’s criteria. Radiological failure: Henning Criteria
Hupperich et al. [14]	2018	3	20	NR	NR	44	12	8.33	Repeat surgical intervention
Steadman et al. [15]	2015	3	19	50	23 (53.5)	192	43	4.65	Repeat surgical intervention
Kang et al. [16]	2015	3	11	48.1	2 (28.6)	16.5	7	14.29	Clinical failure: Barret’s criteria. Radiological failure: Henning Criteria
Steenbrugge et al. [17]	2005	3	11	48.3	10 (53.8)	108	13	23.08	Repeat surgical intervention
Ahn et al. [18]	2004	4	13	46.9	NR	19	9	0	Clinical failure: Barret’s criteria. Radiological failure: Henning Criteria
Noyes et al. [19]	2000	4	13	45	23 (79.3)	33	29	10.34	Repeat surgical intervention

## 5. Discussion

Based on the results of this review, age should not be a contraindication for meniscal repair. Across 13 studies and 316 meniscal repairs, the overall failure rate was 15.6% (range 0–33%). This was higher than found in the previous systematic review on this topic, which reported a failure rate of 10% across 148 patients [3]; it is comparable to that found in a recent systematic review looking at all ages (14.8%) [24]. Surgeons treating symptomatic meniscal tears should therefore consider offering repair to appropriate patients over the age of 40, as even patients significantly older can show excellent functional outcomes. The historic concerns around meniscal repair in older patients was based on the observations that tears more frequently tended to be degenerative in nature, compounded by the presence of degenerative changes within the knee joint. Histological studies have also demonstrated decreased intrinsic and perimeniscal cellularity in those over 40, which is thought to contribute to lower healing potential [24]. This theoretical concern has not been demonstrated in the clinical outcomes of the studies included in this analysis.

When comparing the overall failure rates by age, we found no significant difference in failure rates in patients over and under 40 years of age. Physical activity is a possible explanation for this, as it has been shown to correlate with age. It has been suggested that younger age groups are more likely to engage in athletic activities that cause failure of the repair, for example, in sports that require significant pivoting movements [25]. This theory, however, is contradicted by Everhart et al. [26], who compared the outcomes of meniscal root tears in 61 patients over the age of 40 to 164 patients under 40. They found no difference in failure based on age or activity level. Though the group that took part in pivoting sports tended towards increased risk of failure, this was not deemed to be statistically significant (OR 1.29, CI 0.47, 3.51). Sedentary patients across all age groups were at increased risk of failure and had poor functional outcomes. This suggests that active patients should be offered repair regardless of age to achieve optimal outcomes, without any increased concern regarding reoperation.

Though the review demonstrated low failure rates in those over 40, it should be noted that all studies were retrospective in nature. Patients undergoing repair were actively selected by the treating surgeon. Patients with advanced degenerative changes preferentially underwent meniscectomy in two studies [10,13], and three other studies entirely excluded patients with advanced arthritic changes [11,12,21]. This demonstrates a clear selection bias. However, this does not necessarily undermine the findings of the study, as it demonstrates the importance of patient selection in justifying the outcomes of repair. Data stratifying the impact of degenerative changes on meniscal repair outcomes are limited, as patients in this context would be more likely to undergo total knee replacement as their definitive management [27]. This is both because their broader degenerative changes may be contributing to their symptoms, and coronal malalignment can also be corrected. This review demonstrates older patients with mild degenerative changes should be considered for meniscal repair. Concomitant malalignment should, however, be addressed at the time of surgery as well as through high tibial osteotomy.

Only two studies reported conversion to TKA [10,11], for two cases in total. As studies were retrospective, risks of TKA conversion may have been under-reported. The overall conversion to TKA following meniscal repair was reportedly as low as 0.01%, suggesting that conversion to TKA should not necessarily be a concern in the absence of degenerative changes [28].

Meniscal root tears have been shown to have significantly higher conversion to total knee arthroplasty even after repair (between 9 and 30%) [29,30]. Despite this, an initial repair was still shown to lower the risk of subsequent osteoarthritis and be cost-effective when compared to repair and nonoperative management. Root tears effectively defunction the entire meniscus, and we excluded them from our pooled analysis; they have been demonstrated to have worse outcomes across all age groups and require a notably different operative technique to repair. In spite of this, these reviews have demonstrated that patients would benefit from root repair over meniscectomy.

Other pathologies have also been suggested to impact failure rates. Where it was reported, we found the failure rate in ACL-deficient knees to be 13%. This is similar to what has been reported elsewhere in the literature for all age groups, suggesting that patients over the age of 40 are not at increased risk of failure in the context of an ACL-deficient knee. The failure rates with concomitant ACL reconstruction (ACLR) are significantly lower, at 8.5%. This suggests that patients should be offered concomitant ACLR where feasible; however, the failure rate with meniscus repair alone is not unacceptably high. Thus, repair should be offered even if ACLR is not feasible. The lower failure rate in ACLR is thought to be due to both the biomechanical augmentative effect of the ACL and the favorable biological environment created within the knee joint during tunnel preparation [30]. Further studies are needed to investigate this.

Isolated osteochondral lesions are another important pathology to consider. Degenerative meniscal tears are strongly associated with cartilage defects [31]. Zhu et al. [11] found patients with International Cartilage Repair Society (ICRS) grade 1 and 2 lesions of the medial femoral condyle had improved outcomes compared to those with higher-grade lesions. Osteochondral lesions also impact outcomes following meniscectomy, suggesting these lesions should be addressed in any case. Microfracturing has shown good outcomes in dealing with smaller osteochondral lesions. The use of autologous chondrocyte implantation and mesenchymal stem cell therapy have shown promising outcomes in larger defects and may thus be a useful adjunct to meniscal repair [32]. Similar to ACLR, the biological environment created through microfracture drilling may create optimal conditions for healing of the repair. Indeed, patients in Steadman et al.’s [23] study who underwent microfracture had a lower failure rate than patients where no microfracture was performed (1/21 vs. 7/13), though this did not reach the threshold for statistical significance.

This is the first review to report on Patient Reported Outcome Measures (PROMs) for this age group. PROMs are arguably the most important outcome as they are grounded in what the patient experiences. Like the wider literature, patients undergoing meniscal repair had improved functional outcomes when compared to those undergoing meniscectomies (66.2 vs. 88.6 *p* < 0.0001). The MCID for the Lysholm score has been reported as being between 3 and 12.3 for meniscal injuries [33], suggesting that if meniscal repair is feasible in older patients, they would significantly benefit from it as opposed to meniscectomy. The interesting finding of our study is that across two of the studies encompassing 55 patients, patients over 40 had similar Lysholm scores to patients under 40 (87.6 vs. 85.7).

Chronicity of tear is always an important consideration. It has been thought that early repair leads to improved functional outcomes [34]. Studies have shown reduced cellularity beyond 12 weeks from the time of injury, while other authors have demonstrated increased DNA fragmentation and increased cartilage degeneration with increased time following injury [24]. Pujol et al. did find that increased time to repair correlated with increased volume of meniscus removed after failure; however, they did not demonstrate an increased failure rate [35]. Several earlier studies have also shown that good outcomes can be achieved in chronic tears. There is a significant variation in the literature as to what is defined as chronic. In Engler et al.’s [13] study, most of their meniscal repair cohort were chronic tears, which they defined as >6 weeks (23/26). They demonstrated superior outcomes to their meniscectomy group, with similar reoperation rates. Buyukkuscu et al.’s cohort all underwent a period of conservative treatment lasting >8 weeks and demonstrated good outcomes, with a healing rate of 67% based on radiologic criteria. Interestingly, this study showed no correlation between clinical symptoms and MRI findings; only 36% of patients had features suggesting repair failure on MRI showing one or more physical feature, which suggests that MRI may be overly sensitive in identifying repair failure [21]. Degenerative meniscal tears often present later than acute tears, as they are less associated with acute trauma, and patients rarely have knee effusion [36]. Therefore, treating surgeons are more likely to be presented with chronic tears in the older age groups. Based on this, we would recommend that chronicity alone should not be used as a rationale to refrain from repair where feasible.

There remains a lack of consensus in the literature regarding weight-bearing protocols following meniscal repair. Therefore, postoperative protocols did vary amongst the studies. Based on our data, earlier weight-bearing did not seem to impact failure rates. A significant number of studies have demonstrated no detrimental effect of early active mobilization in isolated meniscal repair [26,37]. However, correlations with tear type, size, and repair method are lacking. In the studies included in this review, the data were not reported in a way in which these factors could be analyzed. In the older age group, it is also important to consider how well the patient will tolerate non-weight-bearing. Indeed, the earlier weight-bearing that partial meniscectomy allows may be a factor in deciding what treatment to offer in older patients. It may be that smaller or more peripheral tears are allowed to weight-bear earlier, whereas larger and more complex tears should be protected. At present, more studies are needed to identify optimal rehabilitation protocols for specific tear patterns.

There are several limitations to this study. Firstly, as mentioned earlier, the studies differed in how they defined failure. Some studies defined failure as return to theater, others by ongoing symptoms, and some studies used radiographic outcomes. It may be that some patients had a clinical failure but never represented or underwent further surgery. Equally radiographic failure was defined via MRI, which has been demonstrated to be overly sensitive in diagnosis of repair failure as residual scar tissue may be misinterpreted as failure [38]. The highest failure rate reported in this review was 33% from Buyukkuscu et al. [21], who defined failure based on Henning’s radiological criteria [16]. Equally, in patients where return to theater was used to define failure, most studies did not report whether reoperation was due to failure of the repair itself. Steadman et al. [15] reported only 20% of their patients who required subsequent arthroscopy had failure of the repair itself. Equally, patients may have presented with symptoms but had not met the clinical threshold to require reoperation. This suggest that studies reporting reoperation as the definition of failure should be interpreted with caution.

To address this, we would suggest that future studies report failure based on the following: (1) accepted clinical criteria such as Barrett’s criteria; (2) second-look arthroscopy results; (3) radiological findings. Reporting failure rates along these categories will firstly allow for a more reliable meta-analysis in the future and, secondly, for a more robust correlation to be made between clinical and radiographic outcomes.

The majority of the studies did not report in more detail the nature of the failures such as laterality, repair technique, tear size, nature of tear, or ACL status. All these factors have been shown to influence failure rates; thus, we were unable to stratify outcomes for predictors of failure. There is a need for more consistency in how outcomes are reported to facilitate more robust meta-analysis; however, this is a common limitation of systematic reviews in general.

Rehabilitation protocol did vary significantly amongst the studies, and this was not controlled for tear pattern or concomitant injuries/surgery. This may have influenced the clinical outcomes and failure rates in some studies.

Finally, we had a mean follow-up of 66 months, and while we found no correlation between follow-up time and failure rate, a longer follow-up is likely needed to truly understand the long-term implications of repair in those over 40. The time of failure from index procedure was poorly reported across all studies. A recent meta-analysis in all age groups suggested 86% of failures occur within 24 months and 14% occur in the subsequent 2 years [23].

## 6. Conclusions

Meniscal tears in older patients remain challenging to treat. While there may be additional factors the surgeon must consider before undertaking repairs in older patients, such as co-existing pathology, age alone should not be used as a contraindication to repair. Patients over the age of 40 do not have increased failure rates and have equivalent functional outcomes to those under the age of 40. They also have significantly better outcomes than patients undergoing partial meniscectomy. We recommend meniscal repair should be attempted where feasible and that surgeons take a holistic view of the patient and co-existing pathology. Further studies are needed to stratify specific risk factors for failure in older age groups.

## Figures and Tables

**Table 1:** Meniscus repair characteristics of patients included in systematic review.

Variable	Count (%)
Meniscus:	*n = 258*
Medial	187 (72.5)
Lateral	66 (25.6)
Both	5 (1.9)
Laterality:	*n = 95*
Right	52 (54.7)
Left	43 (45.5)
Tear location:	*n = 89*
Anterior	7 (7.9)
Body	20 (22.5)
Posterior	62 (69.7)
Tear type:	*n = 217*
Radial	14 (6.5)
Vertical	69 (31.8)
Horizontal	60 (27.6)
Oblique	0
Bucket handle	23 (10.6)
Complex/degenerative	51 (23.5)
ACL reconstruction	*n = 75*
Repair technique	*n = 197*
All-inside	63 (32)
Inside-out	130 (66)
Outside-in	4 (2)
